# Identification of Immune-Related lncRNA Pairs and Construction and Validation of a New Prognostic Signature of Colon Cancer

**DOI:** 10.1155/2022/5827544

**Published:** 2022-03-30

**Authors:** Minxian Xu, Qing Li, Jianfang Zhang, Hui Xie

**Affiliations:** ^1^Department of Oncology, Affiliated Hospital (Clinical College) of Xiangnan University, Chenzhou 423000, Hunan Province, China; ^2^School of Medical Imaging, Laboratory Science and Rehabilitation, Xiangnan University, Chenzhou 423000, Hunan Province, China; ^3^Key Laboratory of Medical Imaging and Artificial Intelligence of Hunan Province, Chenzhou 423000, Hunan Province, China; ^4^Department of Physical Examination, Beihu Centers for Disease Control and Prevention, Chenzhou 423000, Hunan Province, China; ^5^Department of Radiation Oncology, Affiliated Hospital (Clinical College) of Xiangnan University, Chenzhou 423000, Hunan Province, China

## Abstract

**Background:**

More and more evidence has shown that immune-related long noncoding ribonucleic acid (irlncRNAs) is a potential prognostic factor for colon cancer. The relevant gene pair pattern can improve the sensitivity of the prognostic model. Therefore, our present study aimed to identify irlncRNA Pairs and construct and validate a new prognostic signature in colon cancer.

**Methods:**

We downloaded the expression matrix of mRNA and lncRNA of patients with colon cancer and their clinical information from the public TCGA database. We obtained immune genes from the ImmPort database. Coexpression analysis was performed to identify irlncRNAs. We built an irlncRNA pair matrix by comparing the expression levels of each lncRNA pair in a cycle. Univariate Cox regression analysis, LASSO penalized regression analysis, and multivariate Cox regression analysis were performed to determine the final variables to construct the prognostic risk score model (a new signature). We draw the receiver operating characteristic (ROC) curves of the signature and clinical characteristics and determine the optimal cutoff value by the optimal Akaike Information Criterion (AIC) value. Based on the optimal cutoff value of the ROC curve of the signature, colon cancer patients were divided into the high- and low-risk groups. Then, the signature was evaluated by clinicopathological features, tumor-infiltrating immune cells, checkpoint-related biomarkers, targeted therapy, and chemotherapy.

**Results:**

We identified 8 lncRNA pairs including AC103740.1|LEF1-AS1, LINC02391|AC053503.5, WWC2-AS2|AL355916.2, AC104090.1|NEURL1-AS1, AC099524.1|AL161908.1, AC074011.1|AL078601.2, AL355916.2|LINC01723, and AP003392.4|LINC00598 from 71 differently expressed irlncRNAs. We constructed a prognostic risk score model (a new signature) using these optimal eight irlncRNA pairs. ROC curve analysis revealed that the highest AUC value of the signature was 0.776 at 1 year, with the optimal cutoff value of 1.283. Our present study also showed that the constructed signature could accurately identify adverse survival outcomes, prognostic clinicopathological features, and specify tumor invasion status. The expression of immune checkpoint-related genes and chemical drug sensitivity were related to different risk groups.

**Conclusion:**

In our present study, we constructed a new irlncRNA signature of colon cancer based on the irlncRNA pairs instead of the special expression level of lncRNA. We found this signature had not only good prognostic value but also certain clinical value, which might provide a new insight into the treatment and prognosis of colon cancer.

## 1. Introduction

Colon cancer is the second most common cancer in women and the third most common cancer in men worldwide [1]. Its morbidity and fatality both rank fourth among all malignant tumors in China, and there are approximately 360,000 new cases each year in China [2]. Although surgical techniques have made great progress, adjuvant chemotherapy is still an important part of the comprehensive treatment of colon cancer after radical resection, especially for patients with colon cancer in stages III and IV. In addition, recently, herbal medicines, especially allium extracts, have benefited patients as an additional treatment modality in the treatment of colon cancer [3]. Despite this, 80% of colon cancer patients still relapse within three years after radical resection of the primary tumor and postoperative adjuvant chemotherapy [4], suggesting that a considerable proportion of patients do not seem to benefit from the adjuvant chemotherapy after radical resection or benefit less. In recent years, tumor immunotherapy has gradually become a beneficial treatment method by activating the immune system to produce antitumor effects [5]. Especially, its great success in treating melanoma [6] has brought great inspiration to the treatment of other types of tumors. A study by the Netherlands Cancer Institute found that patients with early- to mid-stage colon cancer without distant metastasis could benefit from short-term neoadjuvant immunotherapy [7]. However, some studies have shown that inhibitors of PD-1/−L1 or CTLA have not yet shown relevant efficacy in unselected colorectal cancer [8]. Therefore, the choice of drugs for colon cancer is very challenging.

Long noncoding RNA (lncRNA) is more than 200 nucleotides in length and is a type of noncoding RNA (ncRNA) [9]. It has been reported that lncRNA plays a very important role in the occurrence and development of malignant tumors such as colon cancer [9], and lncRNA plays a very pivotal role in the immune system and has become the focus of immunology [10]. Its abnormal expression can affect the occurrence, development, and prognosis of a variety of immune system diseases [11]. Jiang et al. found that lnc-Lsm3b can inhibit the activity of RIG-I and play a negative feedback regulation role in the late stage of the immune response, which clarifies the relationship between human lncRNA and the immune system [12]. There are also many studies using immune-related lncRNA to predict the prognosis of cancer patients [13–15]. Recently, there have also been some studies on the prognosis of colon cancer based on immune-related lncRNA [16, 17]. In a study of other scholars, it is found that using the new function of lncRNA-mRNA to study the prognosis of esophageal squamous cell carcinoma has obtained very good results [18]. In our research, we took the lead in using immune-related lncRNA-mRNA to study the prognosis of colon cancer and further analyzed the association between immunity and drugs in colon cancer.

## 2. Materials and Methods

### 2.1. Identification of Immune-Related lncRNA

We downloaded the mRNA and lncRNA expression matrix and related clinical information of colon cancer patients from the public database The Cancer Genome Atlas (TCGA) (https://tcga-data.nci.nih.gov/tcga/). We obtained the related immune genes from the ImmPort database (http://www.immport.org). We extracted the colon cancer immune-related mRNA expression matrix from the mRNA expression matrix using R software. We extracted the immune-related lncRNAs (irlncRNAs) expression matrix by the correlation between immune genes and lncRNAs, and the selection criteria were as follows: correlation coefficient >0.4 and *P* value <0.001. Finally, we used the “limma” package of the R software to screen out differentially expressed immune-related lncRNAs (DEirlncRNAs) and the screening criteria were |logFC| ≥ 1 and the *P* value <0.05 (FC: fold change, and *P* value was corrected by Benjamini–Hochberg FDR). Subsequently, we used the “ggplot2” package of the R software to plot the heat map of the differential lncRNA expression.

### 2.2. Constructing the DEirlncRNA Pairs Matrix

Based on the expression level of each lncRNA pair, we constructed the expression matrix of DEirlncRNA pairs. In each lncRNA pair of colon cancer patients, if the expression level of the first lncRNA was higher than the expression level of the second lncRNA, it was marked as 1; otherwise, it was marked as 0. Finally, an expression matrix of 1 or 0 was obtained. The ratio of 0 or 1 in each lncRNA pair was between 0.2 and 0.8. Beyond this range, it was not suitable for predicting the patient's prognosis and must be deleted.

### 2.3. Constructing the DEirlncRNAs Risk Score Model

Combined with the clinical information of colon cancer patients, we performed univariate Cox regression analysis on DEirlncRNA pairs and screened out the DEirlncRNA pairs with survival significance. The screening criterion was *P* value <0.05. In order to further converge the prognostic DEirlncRNA pairs, we performed the Least Absolute Selection Operator (LASSO) regression analysis on the results of univariate Cox regression analysis. In LASSO regression analysis, DEirlncRNA pairs with a frequency greater than 100 were selected with the 10-fold cross-validation and 1000 bootstrap samples methods, and then the selected DEirlncRNA pairs were subjected to Cox proportional hazard analysis. Univariate Cox analysis was performed using the “survival” package of R software, and LASSO analysis using the “glmnet” package of R software. Then, we used the “pROC” package of R software to draw a receiver operating characteristic (ROC) curve of 1, 2, and 3 years and calculated area under the curve (AUC) values with the “ROCR” package of R software. We used the formula RiskScore = ∑_*i*=1_^*n*^*βiSi* to calculate the risk score of each patient, where *β*i is the regression coefficient, and Si is the expression value of DEirlncRNA. We divided patients into high- and low-risk groups, and the optimal cutoff value was determined by the optimal Akaike Information Criterion (AIC) values. Our present study took the maximum inflection point of the 1-year ROC curve as the risk cutoff point.

### 2.4. Constructing the Clinical Prognostic Signature of the Risk Model

In order to further verify the effectiveness of the risk score cutoff point, our present study performed Kaplan–Meier analysis on the high- and low-risk groups to explore the difference in survival and used the survival curve for visualization. Kaplan–Meier analysis was performed using “survival” package of R software. The impact of various factors on the survival of colon cancer patients was observed through risk scores, clinical characteristics such as clinical stage, age and gender, and 1-year ROC curve. Then, the Wilcoxon rank test or *χ*^2^ test was used to analyze the difference between the risk model and clinical characteristics. Finally, we performed univariate and multivariate Cox regression analysis by “survival” package of R software to further explore whether the risk score can be used as an independent prognostic predictor.

### 2.5. Estimation of Tumor-Infiltrating Immune Cells

CIBERSORT is a deconvolution algorithm for the expression of immune cell subtypes based on linear support vector regression. In our present study, we used the CIBERSORT algorithm to calculate the immune cell subtype infiltration score of the included samples. In terms of immune-related analysis, 22 immune cell-related infiltrating score and 28 immune-related pathways were determined by single-sample gene set enrichment analysis (ssGSEA) using the “gsva” package of R software. The signed-rank Wilcoxon test was used to compare the immune infiltration and pathways between the high-risk group and the low-risk group, and *P* < 0.05 was used as a significant threshold. Spearman's correlation test (*P* < 0.05) was performed to assess the correlation between immune cell subtype infiltration rate and the risk score. Kaplan–Meier survival analysis was performed on 22 immune cell-related infiltrating score and 29 immune-related pathways based on the risk score model to further study the correlation between immune cell infiltration and the risk score and prognosis. Kaplan–Meier survival analysis was performed by the “survival, survminer” packages of R software.

### 2.6. Expression of Four Immunosuppressive Molecules

In our present study, we compared the expression levels of immunosuppressive molecules including CTLA4, PDCD1, and LAG3 between high- and low-risk groups using the “ggpubr” package of R software.

#### 2.6.1. Prediction of Drug Response

We used “pRRophetic” package of R software to evaluate the chemotherapeutic drug sensitivity with the half-maximum inhibitory concentration (IC50). We downloaded the drug sensitivity data and gene expression profile data of colon cancer cell lines from the Genomics of Drug Sensitivity in Cancer (GDSC, https://www.cancerrxgene.org) database. Wilcoxon's signed-rank test was used to compare the difference of IC50 between high- and low-risk groups in the risk model.

## 3. Results

**Figure 1:**
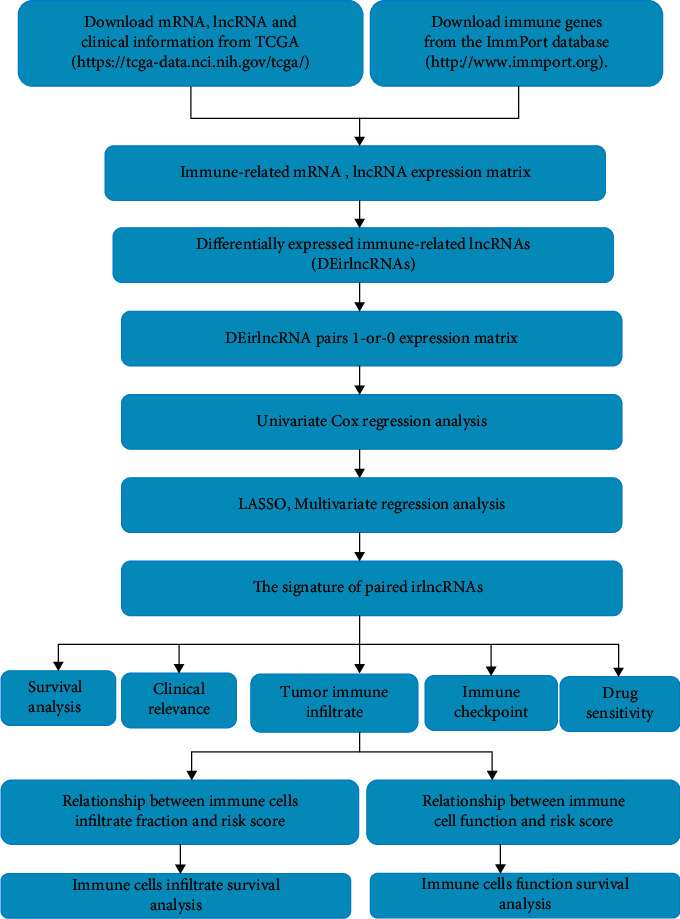
The flowchart of our research.

The flowchart of our research is shown in Figure 1. There were a total of 521 samples in our present study, including 480 colon cancer samples and 41 normal samples (both from the TCGA database). Moreover, 2843 immune genes were downloaded from the ImmPort database (Supplementary data 1).

### 3.1. DEirlncRNAs in Colon Cancer Database Samples

**Figure 2:**
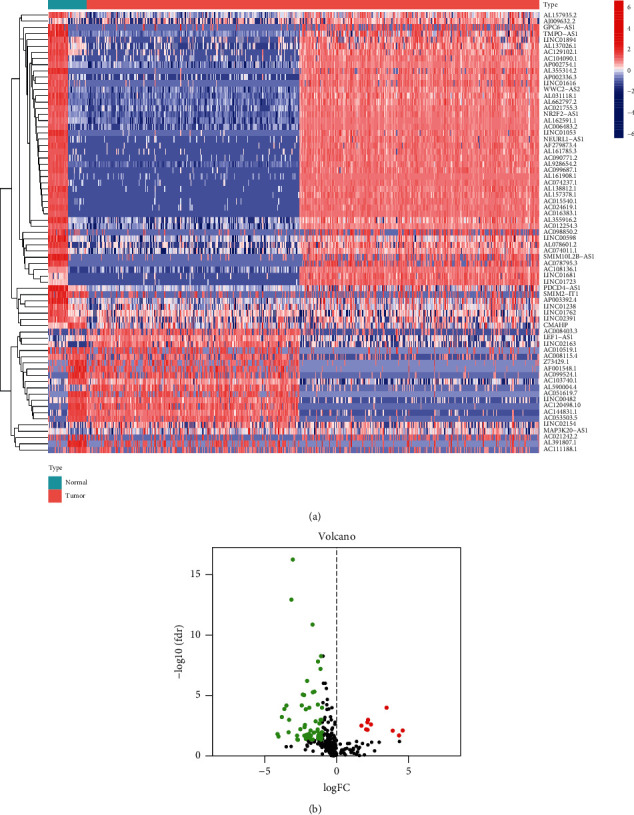
(a) Heatmap of the 71 upregulated and downregulated DEirlncRNA in colon cancer. (b) Volcano plot representing the DEirlncRNA of colon cancer. Upregulated DEirlncRNAs were represented by red dots, and downregulated DEirlncRNAs were represented by green dots.

A total of 1,342 immune-related gene mRNA expression matrices (Supplementary data 2) and 694 immune-related lncRNA expression matrices (Supplementary data 3) were extracted from colon cancer samples. Immune-related lncRNA expression differentiation analysis found that the expression of 71 immune-related lncRNAs had significant differences (|logFC| > 1, adj. *P* < 0.05). The DEirlncRNAs in colon cancer are illustrated in the corresponding heat map (Figure 2(a)) and volcano plot (Figure 2(b)).

### 3.2. Establishment and Validation of the Risk Score Model

**Figure 3:**
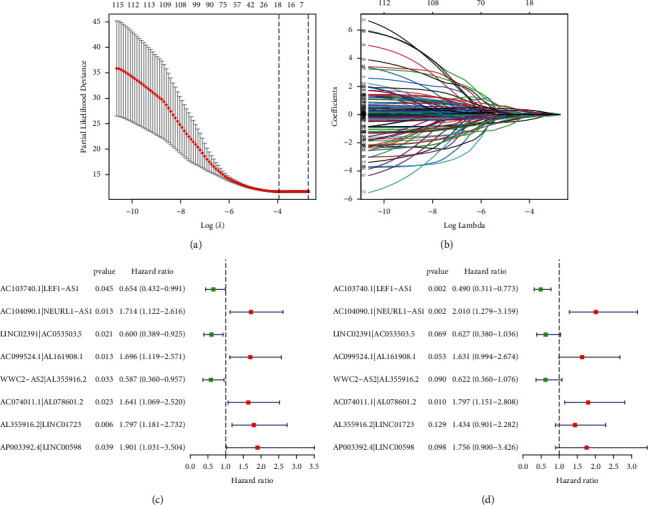
Construction of an irlncRNA signature. (a) *λ* selection by 10-fold cross-validation. The value 0.019 was chosen for *λ* by 10-fold cross-validation with the minimum criteria. (b) Processes of LASSO Cox model fitting. (c) Univariate and (d) multivariate analysis of the influence of DEirlncRNA pairs in colon cancer patients.

**Figure 4:**
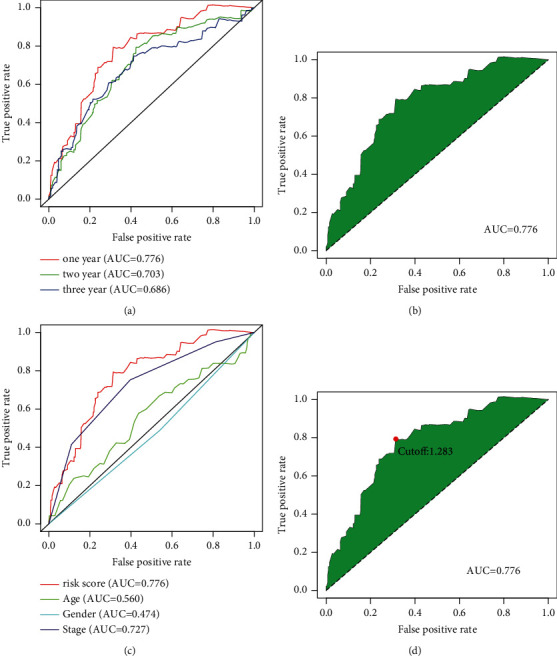
The prediction performance of the receiver operating characteristic (ROC) curves for the model signature. (a) ROC curves show 1-year/2-year/3-year curves and AUC. (b) ROC curve with the optimal AUC value within three years. (c) ROC curves for the comparison of clinical characteristics and signature. (d) The optimal cutoff point plots generated by AIC.

After filtering the DEirlncRNA 1-or-0 matrix, we finally identified 1550 pairs of lncRNA. The univariate Cox regression and LASSO analysis identified 8 lncRNA pairs to construct the risk model (Figure 3(a) and 3(b)), where hazard ratios (HRs) of AC103740.1|LEF1−AS1, LINC02391|AC053503.5 and WWC2−AS2|AL355916.2 were less than 1, while hazard ratios (HRs) of AC104090.1|NEURL1−AS1, AC099524.1|AL161908.1, AC074011.1|AL078601.2, AL355916.2|LINC01723, and AP003392.4|LINC00598 were greater than 1 (Figure 3(c)). The Cox multivariate regression analysis revealed that AC103740.1|LEF1−AS1 (*P*=0.002; HR, 95% CI = 0.490 [0.311–0.730]), AC104090.1|NEURL1−AS1 (*P*=0.002; HR, 95% CI = 2.010 [1.279–3.159]), and AC074011.1|AL078601.2 (*P*=0.010; HR, 95% CI = 1.797 [1.151–2.808]) were considered to be independent significant prognostic factors for colon cancer (Figure 3(d)). The predicted AUC values were 0.776 at 1 year, 0.703 at 2 years, and 0.686 at 3 years, respectively (Figure 4(a)), and the highest AUC value was 0.776 at 1 year (Figure 4(b)). The ROC analysis revealed that the risk score model with 0.776 of AUC value was better fitted than the clinical risk models, including age with 0.560 of AUC value, gender with 0.474 of AUC value, and stage with 0.727 of AUC value (Figure 4(c)). Figure 4(c) showed that the clinical stage also had a higher AUC value, which indicated that the clinical stage might be an important prognostic factor. AIC analysis revealed that the cutoff value of the risk score was 1.283 (Figure 4(d)). Based on this cutoff value, patients with a risk score higher than 1.283 were included in the high-risk group and patients with a risk score lower than 1.283 were included in the low-risk group.

### 3.3. Survival Analysis of the Model and the Correlations between the Risk Score and Clinical Characteristics

**Figure 5:**
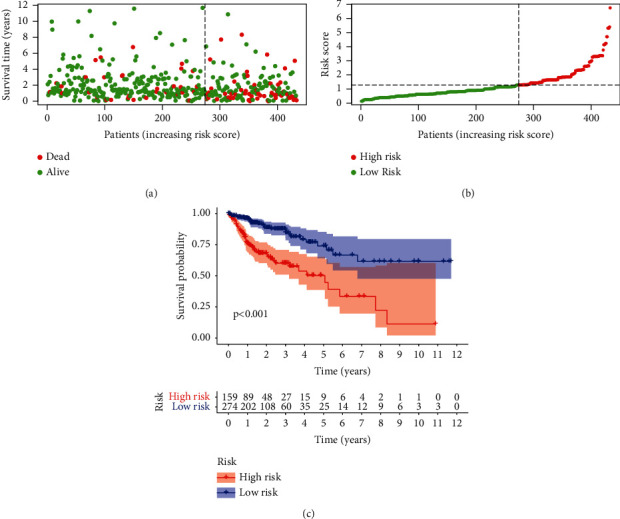
(a) Distribution of risk scores. (b) Distribution of survival outcomes. (c) Survival outcomes based on Kaplan–Meier survival analysis.

**Figure 6:**
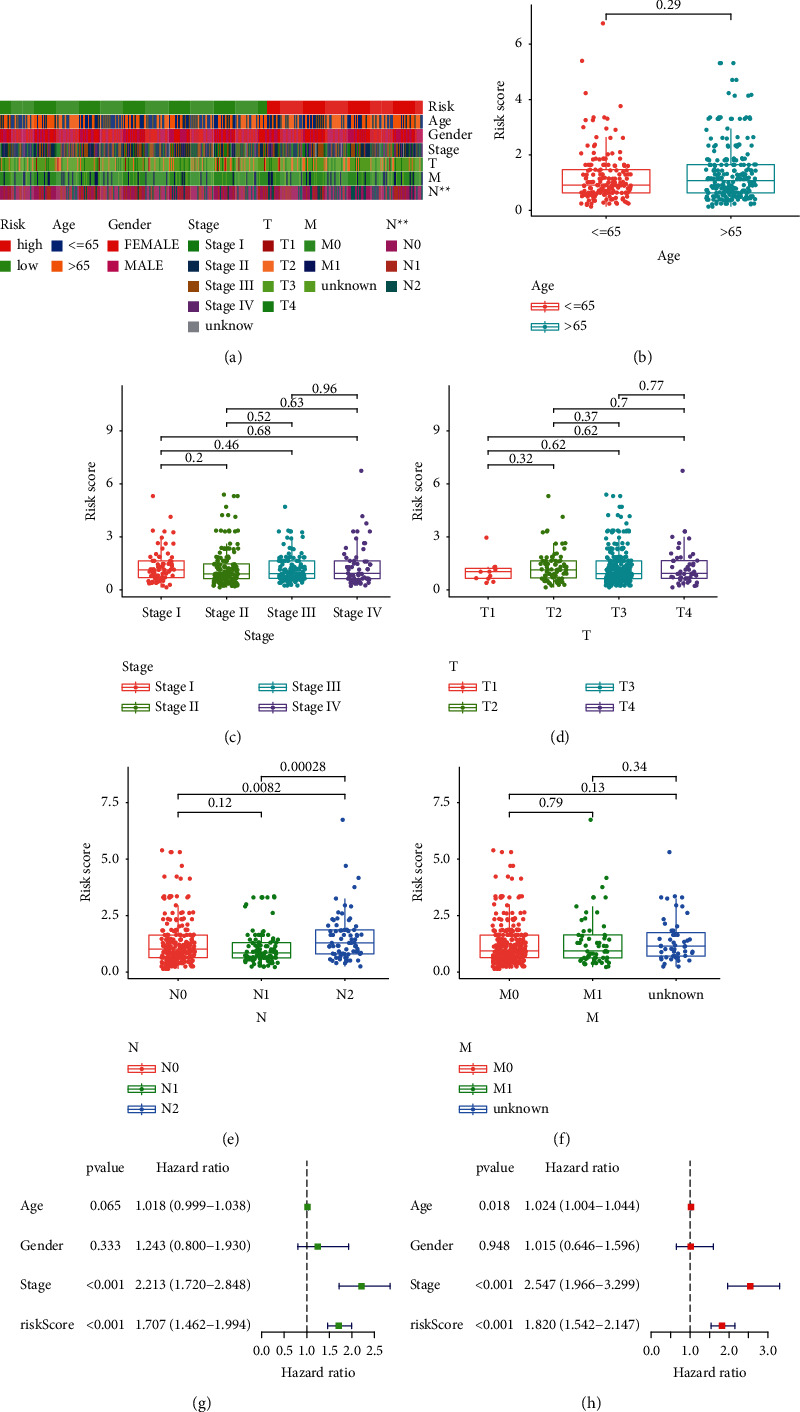
Whether the signature is an independent prognostic factor and relationship between risk score and clinical characteristics is evaluated. (a) Strip chart of clinical characteristics. (b) Age and risk scores; (c) stage and risk scores; (d) T-stage and risk scores; (e) N-stage and risk scores; (f) M-stage and risk scores; (g) univariate and (h) multivariate Cox regression analysis of risk score and clinical characteristics. ^*∗*^represented *P* < 0.05, ^*∗∗*^represented *P* < 0.01, and ^*∗∗∗*^represented *P* < 0.001.

The risk score and survival status of the scatter plot have shown that patients in the low-risk score group have longer survival time (Figures 5(a) and 5(b)). The Kaplan–Meier survival curve analysis showed that patients in the low-risk group had better survival status than those in the high-risk group (Figure 5(c)). The analysis of the correlations between the risk score and clinical characteristics (Figure 6) showed that the risk score was significantly correlated with the N-stage (Figure 6(e)), which implied that patients in the advanced stage were more likely to be at risk than those in the early stage. The results of univariate (Figure 6(g)) and multivariate (Figure 6(h)) Cox regression analysis showed that stage (*P* < 0.001, HR = 2.547, 95% CI = [1.966–3.299]) and risk score (*P* < 0.001, HR = 1.820, 95% CI = [1.542–2.147]) were independent prognostic factors.

### 3.4. Immune Landscape

**Figure 7:**
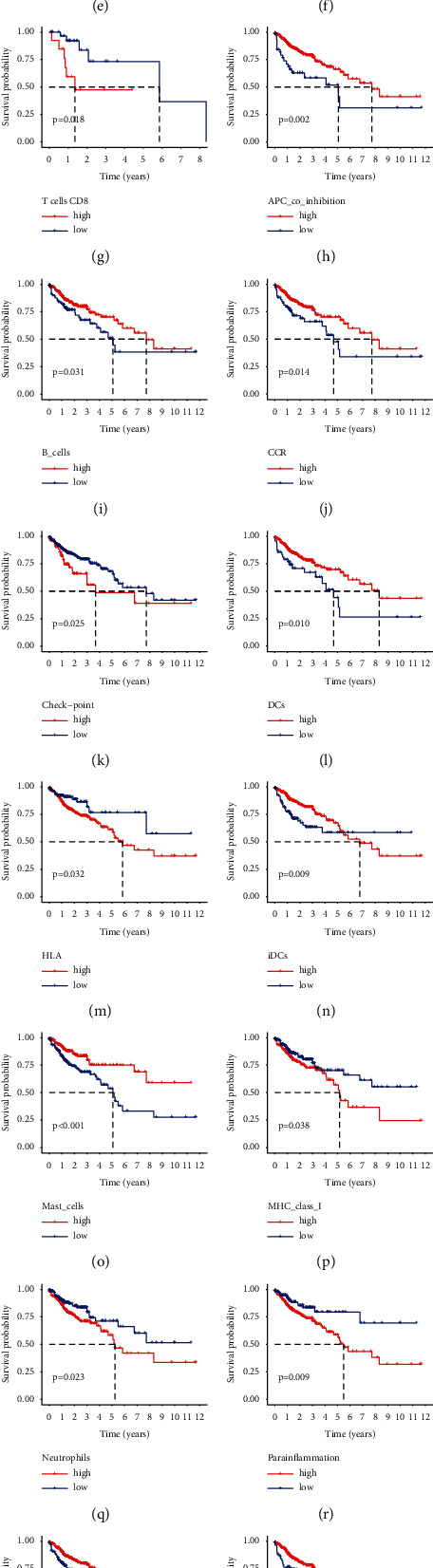
(a) The correlation of immunocyte infiltration fraction with risk score based on high- and low-risk groups. (b) The correlation of immune-related biological processes with risk score based on high- and low-risk groups. (c–g) Immunocyte infiltration cell survival analyses. (h–x). Immune-related biological processes survival analyses.

**Figure 8:**
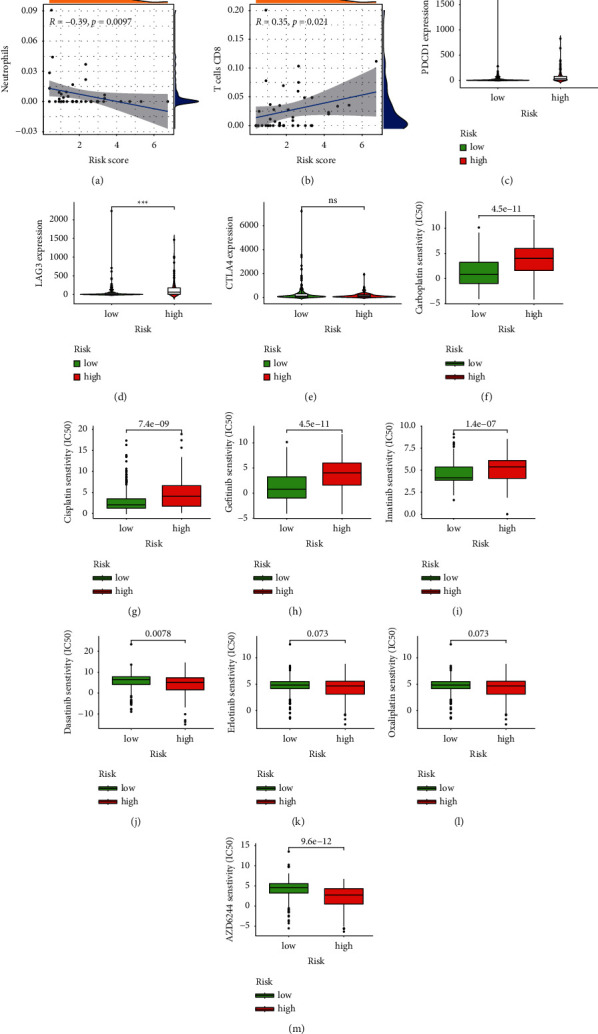
(a–b) The correlation of immunocyte infiltration fraction with risk score. (c–e) PDCD1, LAG3, and CTLA4 expressed levels in high- and low-risk groups. (f–m) The abilities of the risk model to predict drug sensitivity: carboplatin, cisplatin, gefitinib, imatinib, dasatinib, erlotinib, oxaliplatin, and AZD6244. ^*∗∗∗*^represents *P* < 0.001, ^*∗∗*^represents *P* < 0.01, ^*∗*^represents *P* < 0.05, and ns represents not significant.

In our present study, we used the CIBERSORT algorithm to calculate the infiltration of immune cells of colon cancer in the high- and low-risk groups. In order to reveal the differences of the tumor microenvironment (TME) in colon cancer between the high- and low-risk groups, ssGSEA was used to analyze the level of immune cell infiltration and immune cell function in each group, which found that there were statistical differences in the immune cells infiltration rate of B-cells memory and neutrophils between the high- and low-risk groups and the immune infiltration rate of the low-risk group was higher than that of the high-risk group (Figure 7(a)). Immune-related biological processes were significantly different between the high- and low-risk groups except for Th2_cells and TIL (Figure 7(b)). The survival analysis of immune cell infiltration subtypes showed that there was a significant survival difference between the high- and low-risk groups for naive B cells (*P*=0.022; Figure 7(c)), Macrophages M0 (*P*=0.012; Figure 7(d)), resting NK cells (*P*=0.034; Figure 7(e)), plasma cells (*P*=0.021; Figure 7(f)), and CD8 T cells (*P*=0.018; Figure 7(g)). The survival analysis of immune-related biological processes showed that there was significant survival difference between the high- and low-risk groups for APC_co_inhibition (*P*=0.002; Figure 7(h)), B_cells (*P*=0.031; Figure 7(i)), CCR (*P*=0.014; Figure 7(j)), Check-point (*P*=0.025; Figure 7(k)), DCs (*P*=0.010; Figure 7(l)), HLA (*P*=0.031; Figure 7(m)), iDCs (*P*=0.009; Figure), Mast_cells (*P* < 0.001; Figure 7(o)), MHC_class_I (*P*=0.037; Figure 7(p)), Neutrophils (*P*=0.023; Figure 7(q)), Parainflammation (*P*=0.009; Figure 7(r)), pDCs (*P*=0.002; Figure 7(s)), T_cell_co-inhibition (*P*=0.023; Figure 7(t)), T_helper_cells (*P*=0.023; Figure 7(u)), Tfh (*P*=0.032, Figure 7(v)), Th1_cells (*P*=0.006; Figure 7(w)), and TIL (*P*=0.009; Figure 7(x)). There was a significantly negative correlation between the immune cell infiltration of neutrophils and the risk score (*R* = −0.39, *P*=0.0097, Figure 8(a)), while the immune cell infiltration of T cells CD8 was positively correlated with the risk score (*R* = 0.35, *P*=0.021; Figure 8(b)). The high expression of immune checkpoint inhibitor-related genes PDCD1 (Figure 8(c)) and LAG3 (Figure 8(d)) were significantly related to the high risk score, while there was no statistically significant association between the expression level of CTAL-4 and the risk score (Figure 8(e)).

### 3.5. Response to Drug Sensitivity

Our present study assessed the correlations between IC_50_ levels of eight drugs, including carboplatin, cisplatin, dasatinib, erlotinib, gefitinib, imatinib, oxaliplatin, and AZD6244 and the risk score. The higher IC_50_ of carboplatin, cisplatin, gefitinib, and imatinib was significantly correlated with the high risk score (Figures 8(f)–8(i)). The lower IC_50_ of dasatinib, erlotinib, oxaliplatin, and AZD6244 was significantly correlated with the high risk score (Figures 8(j)–8(m)).

## 4. Discussion

As an important participant in biological regulation, lncRNA has been increasingly recognized for its role in predicting prognosis. In the field of colon cancer, many important differentially expressed mRNAs and LncRNAs were selected as prognostic risk factors to predict its prognosis [19, 20]. However, these kinds of research only used the expression of mRNA and lncRNA as the research object. In our present study, we took irlncRNAs pairs as the research object and then used bioinformatics methods to screen the key irlncRNAs pairs as predictors of the prognosis model for colon cancer patients. In our present study, a total of 8 irlncRNAs pairs were included in the final model. Among them, AC103740.1|LEF1-AS1, LINC02391|AC053503.5, and WWC2-AS2|AL355916.2 are prognostic protection factors (HR < 1). Except for LEF1-AS1, the other above lncRNAs were discovered for the first time. LEF1-AS1 is a tumor-promoting factor in gliomas [21] and has a strong carcinogenic effect in oral squamous cell carcinoma [22], and the overexpression of LEF1-AS1 leads to the apoptosis and proliferation of lung cancer cells [23]. However, AC104090.1|NEURL1-AS1, AC099524.1|AL161908.1, AC074011.1|AL078601.2, AL355916.2|LINC01723, and AP003392.4|LINC00598 were prognostic risk factors (HR > 1). Moreover, these lncRNAs were found for the first time as prognostic factors in colon cancer. The ROC curve of the prognostic model constructed by the above eight lncRNAs pairs indicated that the model had high reliability (AUC = 0.776). The results of univariate Cox regression analysis showed that the stage and signature were very important for the prognosis of colon cancer patients. In the multivariate Cox regression analysis, stage and signature were independent prognostic factors. Therefore, the prognostic signature of this research might provide individualized prognostic prediction, diagnosis, and treatment for colon cancer patients.

The results of the ssGSEA analysis suggested that the immune cell infiltration subtypes of B cells memory and neutrophils had higher immune infiltration fraction in the low-risk group than that in the high-risk group, with statistical differences. There were statistically significant differences in the immune-related biological processes, except for Th2cells and TIL, between the high- and low-risk groups. As we all know, B-cells memory and neutrophils mainly secrete IL-1*β* [24], and it has been reported that IL-1*β* can promote the growth of colon cancer cells by activating Wnt signaling [25]. Kaler P et al. confirmed that IL-1*β* can induce Wnt signaling and tumor cell growth by the inactivation of GSK3*β* [26], thereby causing the immune escape in patients with colon cancer, which was also indirectly confirmed by the negative correlation between immune cell infiltration fraction and the risk score in our present study (the low-risk group has a higher neutrophil immune infiltration rate than the high-risk group). The survival status of macrophages M0 in the high-risk group was better, and the immune infiltration rate of Macrophages M0 cells in each sample was the highest. Macrophages M0 cells in colon cancer may be transformed into immunosuppressive and protumor Macrophages M2 cells in the TME [27]. At the same time, they can also affect the progression of colon tumors by promoting tumor angiogenesis and lymphangiogenesis [28]. Macrophages M0 cells may participate in these processes through the intermediary of IL-1*β* [29]. Some studies have also shown that IL-1*β* secreted by neutrophils can attract macrophages, thereby releasing much more IL-1*β* [30]. Our results showed the survival time of macrophages M0 and M1 in the high-risk group was better than that of the low-risk group. The immune system plays a key role in tumor surveillance and prevention. According to the production of cytokines, the immune response is divided into cell-mediated response and body fluid-mediated response. Th1_cells and Th2_cells cross-regulate and inhibit each other by secreting cytokines. Th1/Th2 imbalance is one of the root causes of tumor occurrence or development [31]. In our present study, the humoral immune response of Th2_cells did not show any difference, the reason for which might be that TNF-*α* is secreted by Th1_cells inhibited the activation of Th2_cells [32, 33]. This suggested that in the tumor microenvironment of colon cancer, Th1 cells were dominant and inhibited the humoral immune response and tumor cells in colon cancer patients might easily escape the surveillance of the immune system so that the body cannot prevent an effective antitumor response. A study on animals showed that Th2_cells and related cytokines were involved in the occurrence and development of tumors via many mechanisms, such as macrophages and myeloid-derived cell activation [34, 35].

Immune checkpoint inhibitors, such as those targeting the PD1/PDL1 axis, have shown moderate activity in combination with other therapies to treat colon cancer [36, 37]. A phase II clinical trial of pembrolizumab found that immune checkpoint blockade is a more effective treatment for colon cancer with microsatellite instability (MSI) [38], and mismatch repair (MMR) status can predict the clinical benefit of pembrolizumab, which is shown to be an MMR of the colon and increased cancer response [38]. In the latest investigation on 78 patients, the ORR of pembrolizumab in MSI-H/dMMR colorectal cancer patients was 52%, and the ORR in MSI-H noncolorectal cancer patients was 54% [39]. Nivolumab was also proved to have the antitumor activity in MSI-H/dMMR colorectal cancer [40]. However, accurately identifying patients who respond to immunotherapy remains a huge challenge. Therefore, it is very important to screen out suitable biomarkers for the identification of patients undergoing immunotherapy. It has been reported that the expression level of immune checkpoint genes is related to the prognosis of cancer patients [41]. In this study, we found that signature was significantly correlated with the expression levels of many immune checkpoint genes in colon cancer, which indicated that the signature might have value in predicting the response of colon cancer patients to immunotherapy. In our present study, we found this feature was significantly correlated with PDCD1, LAG3, and IL-10 but not significantly correlated with CTLA4. This discovery greatly improves the immune landscape of our model signature. More importantly, our model not only implies that carboplatin, cisplatin, gefitinib, and imatinib in the high-risk group were significantly correlated with higher IC_50_ but also implies that dasatinib, erlotinib, oxaliplatin, and AZD6244 in the high-risk group were significantly correlated with lower IC_50_. This indicated that our model signature may help clinical medication.

In our present study, we constructed a new irlncRNA signature based on the irlncRNA pairs, which did not depend on the specific expression level of lncRNA. Further comprehensive analysis confirmed that this prognostic model might be used as an independent prognostic factor for colon cancer patients and presented a rich immune landscape.

## Data Availability

The datasets used and/or analyzed during the current study are available from the corresponding author upon reasonable request.

## Supplementary Materials

Supplementary Materials Supplementary data 1: 2843 immune genes were downloaded from the ImmPort database. Supplementary data 2: a total of 1,342 immune-related gene mRNA expression matrices. Supplementary data 3: 694 immune-related lncRNA expression matrices.
